# Persistent Misunderstandings in Rehabilitation Medicine in India: A Call for Redefinition

**DOI:** 10.7759/cureus.103138

**Published:** 2026-02-06

**Authors:** Anurug Biswas

**Affiliations:** 1 Physical Medicine and Rehabilitation, Santiniketan Medical College and Hospital, Bolpur, IND

**Keywords:** disability management, early rehabilitation, multidisciplinary care approach, physical medicine and rehabilitation (pm&r), quality of life (qol)

## Abstract

Physical Medicine and Rehabilitation (PMR) is often misunderstood in India and is seen only as physiotherapy or a supportive service rather than a full clinical specialty. This misconception may lead to late referrals, preventable complications, and poor functional outcomes for people living with disability. PMR is not just about exercises; it is physician-led and focused on diagnosis, medical management, and coordination of multidisciplinary care. Early rehabilitation is beneficial for recovery, but PMR remains marginalized in acute care and medical education. The non-mandatory status of PMR as a subject for medical graduates in India has led to limited awareness of this discipline among future doctors. With disability affecting a large number of people in India, redefining PMR as a core specialty is urgent. Recognizing its role in improving function, participation, and quality of life is essential to move beyond survival as the only measure of success and to ensure better care for millions who need it, a priority underscored in this editorial.

## Editorial

Misconceptions in Physical Medicine and Rehabilitation

Despite significant advances in acute medical and surgical care, long-term functional outcomes for patients with disability continue to lag behind expectations. One major contributor to this gap is the persistent misunderstanding of Physical Medicine and Rehabilitation (PMR) as a supportive or optional service rather than a core clinical specialty. PMR is still frequently equated with physiotherapy alone, overlooking its diagnostic, prognostic, and leadership roles in comprehensive disability management. This misunderstanding has negative consequences for patient outcomes, quality of life (QOL), health expenditure, and healthcare systems.

Late referrals

Across many healthcare settings in India, PMR involvement occurs only after preventable complications such as contractures, pressure injuries, chronic pain, or long-term dependency have already developed. Evidence consistently shows that early rehabilitation improves functional outcomes, shortens hospital stay, and reduces secondary complications [1,2]. Yet PMR is often excluded from acute care pathways, reflecting a system that prioritizes survival over function and QOL. This delayed referral pattern reinforces disability and poor outcomes rather than preventing them [3].

Missing the role of PMR as a care coordinator

PMR plays a vital role in bringing together multidisciplinary rehabilitation teams consisting of physiotherapy, occupational therapy, nursing, psychology, orthotics, social services, and other medical specialties to provide coordinated, patient-centered care. When PMR is marginalized or neglected, care becomes fragmented and inconsistent. Successful care coordination models, such as Kaiser Permanente’s integrated rehabilitation and other teams in the United States, demonstrate that multidisciplinary collaboration led by PMR can be both feasible and impactful, reducing fragmentation and improving patient-centered outcomes [4]. A recent scoping review of rehabilitation care models for post-COVID-19 conditions has demonstrated that only coordinated, multidisciplinary approaches can meet the complex, long-term recovery needs of this population [5]. Redefining PMR as a coordinator of care rather than a downstream service provider is essential for achieving meaningful and sustainable rehabilitation outcomes.

Educational and institutional gaps in India

Misunderstanding of PMR begins early in medical education. Undergraduate education and curricula in India provide very limited exposure to rehabilitation medicine, focusing predominantly on acute disease management. Institutional performance metrics frequently emphasize mortality and discharge rates while neglecting functional recovery and quality of life. This structural undervaluation leads to poor referral practices and limits the growth and impact of PMR within healthcare systems [6]. According to Gazette notification no. “MCI-34(41)/2017-Med./169894,” January 2018, PMR was kept optional for opening a medical college with a 50 Bachelor of Medicine, Bachelor of Surgery (MBBS) students per year intake capacity. Later, in October 2020, Gazette notification no. “NMC/MCI 35(1)98-med.(ii)123627” mentioned PMR as a mandatory department required to start the MBBS course in a medical college. In August 2023, Gazette notification no. “U. 11022/3/2023-UGMEB” removed PMR from the mandatory department list for starting the MBBS course. This recent gazette notification hampered the incorporation and growth of PMR as a subject in medical colleges, especially in private medical colleges. There is also considerable variability in PMR postgraduate teaching among medical colleges across different parts of India. Moreover, a large number of medical colleges in India do not have fully functional PMR departments.

PMR is more than physiotherapy

A common misconception is that PMR begins and ends with exercise prescription or physical modalities. In reality, PMR is a physician-led specialty grounded in medical diagnosis, functional assessment, and individualized goal setting. Physiatrists evaluate impairments, activity limitations, participation restrictions, and environmental factors, integrating medical management with rehabilitation strategies. Medical management of complications of acute and chronic diseases, corrective and rehabilitation surgeries, and pain interventions are a few other tools of a physiatrist or PMR specialist. However, the narrow perception of PMR among other physicians and the general population diminishes its role in early clinical decision-making and deprives patients of coordinated, outcome-focused care [7]. A recent study conducted at a North Indian institute revealed that fewer than half of the people visiting the hospital were aware of the available PMR services [8].

Call for redefinition

PMR must be recognized as a core clinical specialty focused on function, participation, and quality of life. Early integration of PMR into acute care pathways, routine use of standardized functional outcome measures, and stronger representation in medical education are urgently needed. Redefining PMR is not about professional identity alone; it is about ethical, patient-centered care that values functional recovery as a primary outcome [1,7]. The continued misunderstanding of Physical Medicine and Rehabilitation delays appropriate care, increases preventable disability, and places unnecessary burdens on patients and families. Modern medicine cannot claim success by survival alone. Functional recovery must be acknowledged as an essential outcome, and PMR must be positioned accordingly at the heart of disability management. There should be provision for early and mandatory referral to PMR in all government and private setups for patients who need rehabilitation. For this to occur, PMR awareness must be strengthened among practicing physicians and medical students by reincorporating it into the MBBS curriculum. Educating patients and caregivers about the department and its role, directly by PMR specialists, can make these services more visible. This increased awareness will strengthen the case for redefining the department’s place in healthcare. In addition, a PMR department should be established in every tertiary care hospital and progressively extended to district hospitals and community health centers across India to ensure access to comprehensive rehabilitation services. According to the National Family Health Survey (NFHS-5, 2019 to 2021), the overall prevalence of disability in India is 4.52%, affecting more than 63 million people [9]. Addressing misconceptions about PMR is essential so that this large population can be served more effectively and with the care it deserves.
